# Endothelial Lineage Differentiation from Induced Pluripotent Stem Cells Is Regulated by MicroRNA-21 and Transforming Growth Factor β2 (TGF-β2) Pathways*

**DOI:** 10.1074/jbc.M113.495531

**Published:** 2013-12-19

**Authors:** Elisabetta Di Bernardini, Paola Campagnolo, Andriana Margariti, Anna Zampetaki, Eirini Karamariti, Yanhua Hu, Qingbo Xu

**Affiliations:** From the Cardiovascular Division, King's College London, British Heart Foundation Centre, London SE5 9NU, United Kingdom

**Keywords:** Endothelial Cell, Induced Pluripotent Stem Cells, MicroRNA, Transforming Growth Factor Beta (TGFbeta), Vascular Endothelial Growth Factor (VEGF)

## Abstract

Finding a suitable cell source for endothelial cells (ECs) for cardiovascular regeneration is a challenging issue for regenerative medicine. In this paper, we describe a novel mechanism regulating induced pluripotent stem cells (iPSC) differentiation into ECs, with a particular focus on miRNAs and their targets. We first established a protocol using collagen IV and VEGF to drive the functional differentiation of iPSCs into ECs and compared the miRNA signature of differentiated and undifferentiated cells. Among the miRNAs overrepresented in differentiated cells, we focused on microRNA-21 (miR-21) and studied its role in iPSC differentiation. Overexpression of miR-21 in predifferentiated iPSCs induced EC marker up-regulation and *in vitro* and *in vivo* capillary formation; accordingly, inhibition of miR-21 produced the opposite effects. Importantly, miR-21 overexpression increased TGF-β2 mRNA and secreted protein level, consistent with the strong up-regulation of TGF-β2 during iPSC differentiation. Indeed, treatment of iPSCs with TGFβ-2 induced EC marker expression and *in vitro* tube formation. Inhibition of SMAD3, a downstream effector of TGFβ-2, strongly decreased *VE-cadherin* expression. Furthermore, TGFβ-2 neutralization and knockdown inhibited miR-21-induced EC marker expression. Finally, we confirmed the PTEN/Akt pathway as a direct target of miR-21, and we showed that *PTEN* knockdown is required for miR-21-mediated endothelial differentiation. In conclusion, we elucidated a novel signaling pathway that promotes the differentiation of iPSC into functional ECs suitable for regenerative medicine applications.

## Teaser

**Background:** Induced pluripotent stem cells (iPSCs) constitute an attractive source of cells for regenerative medicine.

**Results:** MicroRNA-21 mediates endothelial differentiation derived from iPSCs in presence of VEGF.

**Conclusion:** MicroRNA-21 and TGF-β2 signaling pathways regulate iPSC differentiation to endothelial lineage.

**Significance:** Elucidation of the molecular mechanisms underlying microRNA-21-regulated iPSC differentiation might provide the basic information for stem cell therapy of vascular diseases.

## Introduction

Endothelial cells (ECs)^3^ line the blood vessels of the entire circulatory system and represent the barrier between circulating blood and the rest of the vessel wall. The endothelium is a dynamic and heterogeneous organ with secretory, metabolic, synthetic, and immunological functions (1). There are two main mechanisms that lead to blood vessel formation, namely vasculogenesis and angiogenesis. Vasculogenesis is the formation of new vessels from progenitor cells and was historically believed to occur in embryo development, during the formation of the primitive vascular network from angioblasts (2). Angiogenesis is the formation of new blood vessels from pre-existing ones and occurs both during embryonic development and during physiological and pathological conditions in adult life. Angiogenesis in the infarcted tissue occurs through a complex link between extracellular matrix, ECs, and pericytes, in response to an imbalance of angiogenic factors, compared with angiostatic factors in the local environment (3, 4).

Atherosclerosis is a chronic inflammatory disease, started by endothelial dysfunction, that leads to the formation of an atherosclerotic plaque (5, 6). Balloon angioplasty and stenting are routinely used in clinical practice to treat these conditions. However, one of the major issues of stenting is restenosis, leading to the recurrence of symptoms through EC loss and subsequent smooth muscle proliferation and matrix deposition, thus originating luminal narrowing (7). It has been reported that after vascular injury, restenosis development can be prevented through accelerated re-endothelialization by mature ECs, which inhibits smooth muscle migration, proliferation, and neointima formation (8). In the past several years, accumulating evidence suggests that ES cells, a promising source of pluripotent stem cells with unlimited growth and self-renewal abilities, are able to differentiate into ECs *in vitro* and *in vivo* (9). The ethical and immunological problems associated with the use of ES cells have been recently bypassed using induced pluripotent stem cells (iPSCs), because they can be derived from somatic cell population isolated from the patient. In this study, we indeed aimed at understanding the molecular mechanisms of iPSC differentiation into ECs, to define new methods to produce large number of ECs with high purity from iPSCs: iPSC-derived ECs can be used to treat damaged vessels, to avoid restenosis, and to develop tissue engineered vascular grafts, now major challenges for the future of regenerative medicine (10).

miRNAs are single-stranded, noncoding molecules of RNA, 20–25 nucleotides long, able to regulate a wide range of cellular processes by binding to noncoding regions of mRNA (11). Recent studies focused on angiogenesis-associated miRNAs that are involved in ES cells differentiation toward ECs (12). It has been recently reported that miR-21 overexpression in human prostate cancer cells increased hypoxia-inducible factor-1α (HIF-1α) and VEGF expression, thereby inducing tumor angiogenesis (13). On the other hand, miR-21 has been shown to have antiangiogenic functions, targeting *RhoB* in ECs (14) and *TGF*-β*RII* in human adipose-derived stem cells (hADSCs), thus decreasing the tumor vascularity induced by the cells (15). In addition, in endothelial progenitor cells, miR-21 has been found to induce cell senescence and reduce angiogenesis *in vitro* and *in vivo* (16). In addition to the role of miR-21 in angiogenesis, miR-21 has been also shown to induce adipogenic differentiation of hADSCs via targeting *TGF*-β*RII* (17). However, so far there are no studies showing a link between miR-21 and endothelial differentiation from iPSCs.

TGF-β is a multifunctional cytokine that regulates proliferation, migration, differentiation, and survival of many different cell types. Deletion or mutation of different members of the TGF-β family have been shown to cause vascular remodeling defect and absence of mural cell formation, leading to embryonic lethality (18) or severe vascular disorders (19, 20). It has been previously demonstrated that stimulation of hADSCs with TGF-β2 increased the levels of VEGF and interleukin-6, thus promoting the proangiogenic action of the cells in a hind limb ischemia model (15). However, no previous report showed an involvement of TGF-β in endothelial differentiation of iPSCs. In particular, we focused on the study of one of the three known isoforms of TGF-β, TGF-β2. In the present study, we described a novel protocol for iPSC differentiation into ECs, and we elucidated the role of miR-21 in mediating this process, through targeting-specific pathways. We highlighted for the first time, to our knowledge, a connection between VEGF, miR-21/Akt, and TGF-β2 in the endothelial differentiation of iPSCs.

## EXPERIMENTAL PROCEDURES

### 

#### 

##### Materials

Cell culture media, serum, and cell culture supplements were purchased from ATCC, Millipore, Invitrogen, and PAA. Antibodies against VE-cadherin, Flk-1, eNOS, and GAPDH were purchased from Abcam. Antibodies against vWF, Akt 1/2, and Ser(P)-473 Akt were purchased from Santa Cruz. Antibodies against CD31 were purchased from Abbiotec and Santa Cruz. Antibody against PTEN was purchased from New England Biolabs. The secondary antibodies for immunostaining anti-goat Alexa 488, anti-goat Alexa 594, and anti-rabbit Alexa 488, and anti-rabbit Alexa 594 were purchased from Invitrogen. The secondary antibodies for Western blotting were purchased from Dakocytomation.

##### Cell Culture, Differentiation, and Treatment of iPSCs

iPSCs were generated in our laboratory starting from mouse embryonic fibroblasts isolated as stated in Ref. 21, using a similar method stated in Ref. 22. iPSCs were cultured on gelatin-coated flasks (PBS containing 0.04% of gelatin from bovine skin; Sigma) in DMEM (ATCC) supplemented with 10% fetal bovine serum, 100 IU/ml penicillin, and 100 μg/ml streptomycin (Invitrogen); 10 ng/ml recombinant human leukemia inhibitory factor (Millipore); and 0.1 mm 2-mercaptoethanol (Invitrogen) in a humidified incubator supplemented with 5% CO_2_. The cells were passaged every 2 days at a ratio of 1:6. Differentiation of iPSCs was obtained by seeding the cells on type IV mouse collagen (5 μg/ml)-coated dishes in differentiation medium (DM) that contains α-MEM supplemented with 10% FBS (Invitrogen), 0.05 mm 2-mercaptoethanol, 100 units/ml penicillin, and 100 μg/ml streptomycin. The medium was supplemented with 50 ng/ml VEGF or 3 ng/ml TGFβ-2 (Peprotech), and the cells were maintained under these conditions for up to 7 days. 5 μm SMAD3 inhibitor SIS3 (Calbiochem), and 0.1 μg/ml VEGF neutralizing antibody (R&D System) were added to iPSCs cultured for 7 days with TGFβ-2; 1 μg/ml TGFβ-2 neutralizing antibody (R&D System) was added to the cells 24 h after transfection with pre-miR-21 (Pre-21) for further 24 h. The number of iPSCs seeded on collagen IV for the differentiation was 1.3 × 10^4^/cm^2^ (d3), 6.6 × 10^3^/cm^2^ (d5), and 3.3 × 10^3^/cm^2^ (d7).

##### Quantitative RT-PCR

Relative gene expression was determined by quantitative real time PCR, using 2 ng of cDNA (relative to RNA amount) for each sample with the SYBR Green Master Mix in a 20-μl reaction. *Ct* values were measured using ABI Prism 7000 sequence detector (Applied Biosystems). The 18 S ribosomal RNA served as the endogenous control to normalize the amounts of RNA in each sample. For each sample, PCR was performed in duplicate in a 96-well reaction plate (Eppendorf, twin.tec real time PCR plates). The gene was considered undetectable beyond 35 cycles. The primer sets used for this study are as follows: 18 S, forward, 5′-CCAGTAAGTGCGGGTCATAA-3′ and reverse, 5′-CCGAGGGCCTCACTAAACC-3′; VE-cadherin, forward, 5′-AAGAAACCGCTGATCGGCA-3′ and reverse, 5′-TCGGAAGAATTGGCCTCTGTC-3′; CD31, forward, 5′-CAAACAGAAACCCGTGGAGAT-3′ and reverse, 5′-ACCGTAATGGCTGTTGGCTTC-3′; Flk-1, forward, 5′-TGAAATTGAGCTATCTGCCGG-3′ and reverse, 5′-TTTGAAGGTGGAGAGTGCCAG-3′; vWF, forward, 5′-GGCTGTGCGGTGATTTTAACAT-3′ and reverse, 5′-CGTTTACACCGCTGTTCCTCA-3′; and TGF-β2, forward, 5′-CTTCGACGTGACAGACGCT-3′ and reverse, 5′-GCAGGGGCAGTGTAAACTTATT-3′.

##### miRNA Extraction, Reverse Transcription, Preamplification, and TaqMan Quantitative PCR Assay

Extraction of total RNA including miRNA was performed using the miRNeasy mini kit (Qiagen), according to the manufacturer's protocol. miRNA reverse transcription and amplification were performed using the Megaplex reverse transcription primers (rodent pools A and B, v3.0) and the Megaplex PreAmp primers (rodent primers A and B, v3.0) (Applied Biosystems) as previously described (23). TaqMan miRNA assays were used to assess the expression of individual miRNAs as described before (23).

##### miR-21 Transient Transfection

Manipulation of miR-21 levels in iPSCs differentiated in presence of VEGF for 3 days and cultured to 60–70% confluence was performed using Pre-21, or the nontargeting control (Pre-ctrl) (Ambion AB), whereas inhibition of miR-21 was performed using the locked nucleic acid (LNA) inhibitor of miR-21 (LNA-21) or a negative control (LNA-ctrl) (Exiqon). All transfections were performed using Lipofectamine^TM^ RNAiMAX (Invitrogen), in accordance with the manufacturer's protocol.

##### TaqMan miRNA Array

The expression profile of miRNAs in the samples was determined using the rodent TaqMan miRNA arrays A and B (Applied Biosystems), which is a set of two 384-well microfluidic cards (arrays A and B, v3.0). The arrays enable quantification of gene expression levels of 375 miRNAs per pool and are specific to human, mouse, or rat. Five endogenous controls and a negative control were included in each array for data normalization. PCRs were performed using 450 μl of the TaqMan Universal PCR Master Mix No AmpErase UNG (2×) and 9 μl of the diluted preamplification product to a final volume of 900 μl. 100 μl of the PCR mix were loaded to each port of the TaqMan miRNA array. The fluidic card was then centrifuged and mechanically sealed. Real time PCR was carried out on an Applied Biosystems 7900HT thermocycler using the manufacturer's recommended program.

##### Immunoblotting and Immunofluorescent Staining

For Western blot analysis, cell pellets were resuspended in radioimmune precipitation assay buffer and sonicated. The protein concentration was measured by the Bradford method. 20–50 μg of each sample was loaded on 6–8% polyacrylamide gels, and standard immunoblotting procedure was performed using the listed primary antibodies.

Immunostaining was performed as described previously (24). Adherent cells were fixed in 4% buffered paraformaldehyde and permeabilized (in the case of intracytoplasmatic antigens) with 0.1% Triton X prior and blocked in 1% BSA before incubation with primary antibody for VE-cadherin, CD31, vWF, and eNOS.

##### In Vitro and in Vivo Tube Formation Assay

iPSCs were differentiated in the presence of VEGF for 4 days and then transfected with Pre-21, Pre-ctrl, LNA-21, and LNA-ctrl. *In vitro* angiogenesis assays was performed after 48 h as described previously (25); *in vitro* angiogenesis assay was also performed after 7 days of differentiation in the presence of VEGF or TGF-β2 treatment. *In vivo* angiogenesis was performed 48 h after miR-21 transfection by mixing 5 × 10^5^ cells with 200 μl of Matrigel and injecting it subcutaneously in mice (C57BL/6). The cryosections of the Matrigel plugs were stained with CD31 and VE-cadherin antibodies. Immunostaining was assessed by confocal imaging, and capillary density was calculated as the number of capillary number/mm^2^.

##### Mice

All procedures were performed according to protocols approved by the Institutional Committee for Use and Care of Laboratory Animals. All animals used in this study were inbred mice of C57BL/6 background.

##### Luciferase Reporter Assay

For the luciferase reporter assays, 3 × 10^4^ iPSCs cells were seeded in each collagen-coated well of a12-well plate in DM containing VEGF. 72 h later, cells were transfected with the plasmid expressing luciferase under the control of the *PTEN* 3′-UTR (pGL3-PTEN-wt) and the miR-21 inhibitor and its control. As a control, the same experiment was performed with a plasmid expressing luciferase under the control of a mutated and inactive *PTEN* 3′-UTR (pGL3-PTEN-mut). Briefly, 0.33 μg/well of the reporter plasmids (AddGene, Joshua Mendell laboratory (26)) were co-transfected with the miR-21 inhibitor and the inhibitor negative control using jetPRIME® (2 μl/well) (Polyplus-transfection SA) according to the protocol provided. 48 h after transfection, luciferase activity was measured using the dual luciferase assay system (Promega). The firefly luciferase activity of each sample was normalized to *Renilla* luciferase activity.

##### shRNA Lentiviral Particle Transduction

Lentiviral particles were produced by transfecting HEK 293T with shPTEN pLKO.1 plasmid (Sigma Mission) or shTGF-β2 as described previously (27). Knockdown of *PTEN* and *TGF*-β*2* was achieved by infecting iPSCs differentiated for 3 days in presence of VEGF with PTEN and TGF-β2 shRNA lentiviruses, respectively (shPTEN and shTGF-β2). Cells were infected with shPTEN, shTGF-β2, or the nontargeting control (shNT) (10^7^ transfection units/ml) complete growth medium supplemented with 10 μg/ml of polybrene for 16–24 h. The viruses were then removed, and the cells were transfected with LNA-21 and LNA-Ctrl or with Pre-21 and Pre-Ctrl, as described before. Cells were harvested for further analysis after 48 h.

##### Enzyme-linked Immunosorbent Assay

The concentration of the VEGF and TGF-β2 released glycoprotein in the supernatant was detected by VEGF and TGF-β2 ELISA kits (Invitrogen and R&D, respectively) according to the manufacturers' protocol.

##### Statistical Analysis

Data expressed as the means ± S.E. were analyzed with a two-tailed Student's *t* test for two groups or pairwise comparisons. A value of *p* < 0.05 was considered significant.

## RESULTS

### 

#### 

##### VEGF Induces Functional Differentiation of iPSCs toward EC Lineage

**FIGURE 1.**
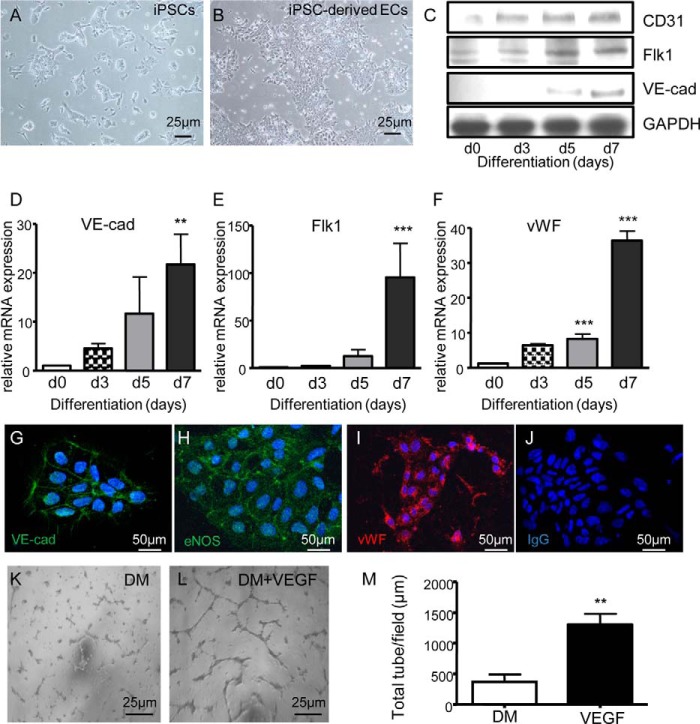
**VEGF induces functional differentiation of iPSCs into ECs.** *A–J*, iPSCs were cultured on collagen IV and in DM supplemented with 50 ng/ml VEGF, followed by morphological analysis (iPSCs undifferentiated on gelatin used as a control (*A*) and iPSC-derived ECs (*B*); *scale bar*, 25 μm), Western blotting (*C*), quantitative RT-PCR assay (*D–F*), (day 0 (*d0*) corresponds to the undifferentiated cells), and immunofluorescence staining (negative controls consisted of cells incubated with IgG followed by secondary antibody; *scale bar*, 50 μm) (*G–J*). The results show the expression levels of the EC markers CD31, Flk1, VE-cadherin (*VE-cad*), vWF, and eNOS. *K–M*, representative images of *in vitro* angiogenesis assay in iPSCs differentiated with or without VEGF for 7 days (DM and DM VEGF; *scale bar*, 25 μm) (*K* and *L*) and total tube length quantification (*M*). The data presented are representative of means ± S.E. (*error bars*) of three independent experiments. **, *p* < 0.01; ***, *p* < 0.001.

We have adapted previous protocol (24) to the differentiation of mouse iPSCs by optimizing concentrations of VEGF and differentiation time points (data not shown). Stimulation of the cells with 50 ng/ml of VEGF rapidly induced a marked change in the morphology of iPSCs, which lost their three-dimensional organization and displayed a flat adherent phenotype (Fig. 1, *A* and *B*). Protein analysis showed a consistent up-regulation of endothelial markers such as CD31, Flk1, and VE-cadherin, starting after 3 and 5 days of differentiation and peaking at day 7 (Fig. 1*C*). Gene expression analysis confirmed this pattern of differentiation for *VE-cadherin*, *Flk1*, and *vWF* (Fig. 1, *D–F*). Furthermore, staining for VE-cadherin, eNOS, and vWF showed the localization of the proteins within the cells and their homogeneous expression in the population (Fig. 1, *G–J*). Finally, the functionality of ECs derived from iPSCs was tested in an *in vitro* angiogenesis assay, showing a significant increase in tubelike structure formation ability upon cell differentiation, as compared with the cells grown in absence of VEGF (Fig. 1, *K–M*). These findings indeed showed that the protocol established allows the generation of a homogeneous and functional population of endothelial cells from iPSCs.

##### Characterization of miRNA Profile in iPSCs Differentiated to ECs

**FIGURE 2.**
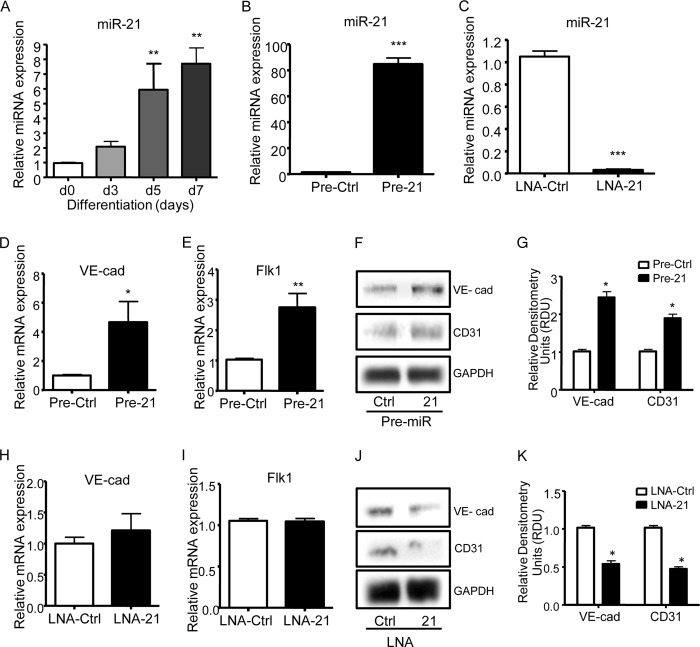
**miR-21 is up-regulated in iPSCs differentiated with VEGF and is able to regulate this process.** *A–C*, iPSCs were differentiated for up to 7 days in the presence of VEGF, followed by TaqMan quantitative PCR assay to analyze the expression of mature miR-21 (*A*). After 3 days of iPSC differentiation with VEGF, the levels of miR-21 were altered by transfecting the cells with precursor of miR-21 (Pre-21), negative control precursor (Pre-Ctrl) and inhibitor of miR-21 (LNA-21), and negative control inhibitor (LNA-Ctrl), followed by TaqMan quantitative PCR assay to assess the levels of mature miR-21 (*B* and *C*). *D–G*, quantitative RT-PCR assay (*D* and *E*) and Western blotting (*F*, representative image; *G*, quantitative analysis) were performed 48 h after miR-21 overexpression. *H–K*, quantitative RT-PCR assay (*H* and *I*) and Western blotting (*J*, representative image; *K*, quantitative analysis) were performed 48 h after miR-21 inhibition. The expression levels of the endothelial markers VE-cadherin (*VE-cad*), Flk1, and CD31 were assessed. The data presented are representative of means ± S.E. (*error bars*) of three independent experiments. *, *p* < 0.05; **, *p* < 0.01; ***, *p* < 0.001.

To investigate the miRNA profile during vascular differentiation of iPSCs, which might be involved in angiogenesis (28), we performed a miRNA array in undifferentiated and differentiated iPSCs for 3 days in the presence of 50 ng/ml VEGF; we obtained two pools of differentially expressed miRNAs (Table 1). Among the miRNAs that were most differentially expressed, we chose to focus on miRNA-21. miR-21 has been previously shown to be involved in angiogenesis (13–16) and adipogenic differentiation of stem cells (17), but its role in stem cell differentiation into endothelial cells remains to be elucidated. Real time PCR confirmed the overexpression of miR-21 starting at day 3 and peaking at days 5 and 7 (Fig. 2*A*). To study its role in differentiation, we transfected the cells after 3 days of culture in the presence of VEGF with miR-21 precursor and the precursor negative control (Pre-21 and Pre-Ctrl; Fig. 2*B*) or miR-21 inhibitor and the inhibitor control (LNA-21 and LNA-Ctrl; Fig. 2*C*) and analyzed the expression levels of endothelial markers. Overexpression of miR-21 significantly induced *VE-cadherin* and *Flk1* gene expression (Fig. 2, *D* and *E*). Consistently, protein analysis confirmed the induction of the protein levels of VE-cadherin and CD31 (Fig. 2, *F* and *G*). Although miR-21 inhibition did not affect the gene expression of the EC markers (Fig. 2, *H* and *I*), it led to a significant decrease in the protein induction of these markers (Fig. 2, *J* and *K*). Even though we should expect a decrease in protein levels derived from RNA levels, the translational efficiency is differentially affected in the inhibitory system. Moreover, these differentiation markers are indirect targets of the miR-21; the effect on their expression is therefore mediated by other pathways.

**TABLE 1:** **MicroRNA array on iPSCs differentiated for 3 days with VEGF and undifferentiated cells** The microRNA arrays were performed on iPSCs differentiated for 3 days on collagen IV and in differentiation medium containing 50 ng/ml VEGF; undifferentiated cells were used as control. The results represent averages of the differential expression between two independent experiments. In the panel are shown 28 and 7 microRNAs respectively up- and down-regulated at least 2-fold relative to the undifferentiated iPSCs. miR-302a#, 34c#, and 92a# represent miR* passenger strands. The results were obtained after normalization with three different endogenous controls (mouse small nucleolar RNA snoRNA202 and 135 and mammalian MammU6). These microRNAs were initially characterized in mouse or rat (mmu, *Mus musculus*; rno, *Rattus norvegicus*).

Target name	snoRNA202	snoRNA135	MammU6
mmu-let-7c	6.7 ± 3.4	4.9 ± 3.2	6.8 ± 1.9
mmu-miR-129–3p	33.9 ± 15	22.6 ± 6.2	39.1 ± 25.9
mmu-miR-133a	7.2 ± 4.4	5.3 ± 3.9	7.2 ± 2.8
mmu-miR-139–5p	3.6 ± 2.5	2.3 ± 1.3	4.2 ± 3.7
mmu-miR-188–5p	3.3 ± 0.5	2.3 ± 0.7	3.5 ± 0.4
mmu-miR-20b	3.1 ± 1.6	2.1 ± 0.7	3.6 ± 2.6
mmu-miR-21	22.7 ± 27.3	17.5 ± 21.8	21.0 ± 23.4
mmu-miR-218	2.9 ± 0.5	2.1 ± 0.7	3.1 ± 0.3
mmu-miR-224	126.5 ± 170.7	98.5 ± 134.3	114.3 ± 151.0
mmu-miR-26b	23.0 ± 13.1	15.1 ± 6.3	26.9 ± 20.7
mmu-miR-29b	145.9 ± 189.9	113.2 ± 150.1	132.7 ± 166.7
mmu-miR-302a	88.1 ± 3.6	61.5 ± 13.4	95.7 ± 20.7
mmu-miR-302b	4,432 ± 4,226	2,820 ± 2,391	5,436 ± 5,862
mmu-miR-302c	27,395 ± 34,072	21,156 ± 27,080	25,158 ± 29,548
mmu-miR-340–5p	7.8 ± 2.0	5.6 ± 2.4	8.3 ± 0.0
mmu-miR-34b-3p	6.6 ± 1.1	4.7 ± 1.1	7.1 ± 0.6
mmu-miR-367	65.9 ± 16.9	44.8 ± 3.6	74.4 ± 37.0
mmu-miR-449a	19.8 ± 12.9	14.5 ± 11.4	19.8 ± 8.6
mmu-miR-685	2.9 ± 0.2	2.0 ± 0.2	3.2 ± 1.1
rno-miR-224	9.3 ± 5.1	6.1 ± 2.4	10.8 ± 8.2
hsa-miR-200b	2.0 ± 0.6	2.0 ± 0.3	2.5 ± 0.6
mmu-miR-212	4.4 ± 1.0	4.3 ± 0.5	5.6 ± 1.6
mmu-miR-2138	6.5 ± 4.5	6.2 ± 3.8	7.1 ± 1.8
mmu-miR-302a#	108.1 ± 46.1	104.7 ± 34.8	129.4 ± 9.8
mmu-miR-34c#	7.0 ± 2.5	6.8 ± 1.7	8.5 ± 1.3
mmu-miR-374–5p	8.9 ± 7.1	8.4 ± 6.1	9.5 ± 3.6
mmu-miR-449b	370.8 ± 512.4	341.1 ± 469.7	326.7 ± 440.3
mmu-miR-92a#	4.1 ± 2.1	3.9 ± 1.7	4.7 ± 0.1
mmu-miR-145	0.3 ± 0.1	0.2 ± 0.1	0.3 ± 0.1
mmu-miR-467e	0.5 ± 0.3	0.4 ± 0.3	0.5 ± 0.2
mmu-miR-669a	0.5 ± 0.2	0.3 ± 0.2	0.5 ± 0.1
mmu-miR-409–3p	0.3 ± 0.4	0.3 ± 0.3	0.3 ± 0.3
mmu-miR-467a	0.3 ± 0.1	0.3 ± 0.1	0.4 ± 0.0
mmu-miR-690	0.4 ± 0.1	0.3 ± 0.1	0.4 ± 0.0
mmu-miR-706	0.4 ± 0.2	0.4 ± 0.2	0.4 ± 0.0

Importantly, miR-21 did not affect EC marker expression in the absence of VEGF stimulation (data not shown); this result indicates that VEGF stimulation is required to initiate the differentiation process toward an endothelial progenitor cell population in which miR-21 induction is able to further induce the EC marker up-regulation. Moreover, no effect of miR-21 overexpression was detected for smooth muscle markers and other mesoderm, endoderm, and ectoderm lineage markers (data not shown).

**FIGURE 3.**
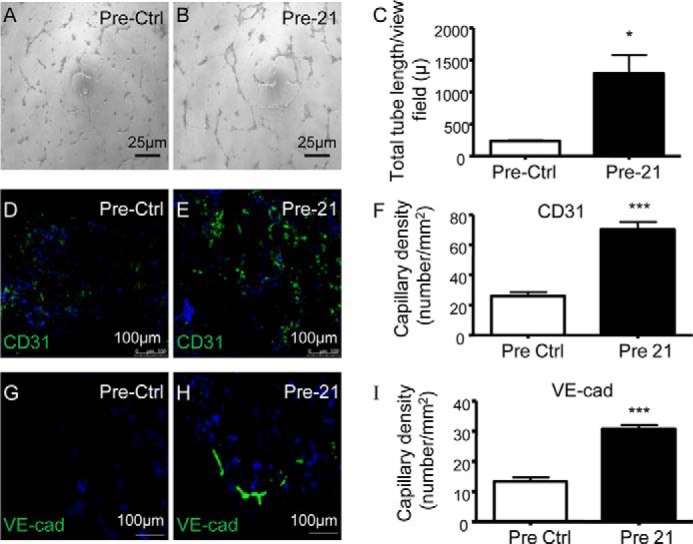
**miR-21 overexpression induces functional iPSC differentiation into ECs *in vitro* and *in vivo*.** *A–I*, iPSCs differentiated for 4 days in the presence of VEGF were transfected with miR-21 precursor (Pre-21) and the negative control (Pre-Ctrl). *A–C*, representative images of *in vitro* angiogenesis assay (*scale bar*, 25 μm) (*A* and *B*) and tube length quantification (*C*). *D–I*, representative images (*scale bar*, 100 μm) of *in vivo* angiogenesis assay and quantification of capillaries positive for the endothelial markers VE-cadherin (*VE-cad*) (*D–F*) and CD31 (*G–I*). The data presented are representative of means ± S.E. (*error bars*) of three independent experiments. *, *p* < 0.05; ***, *p* < 0.001.

**FIGURE 4.**
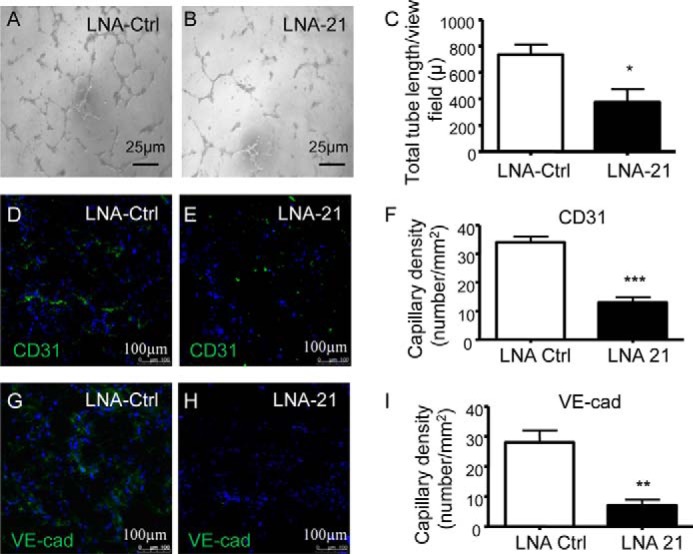
**miR-21 inhibition decreases functional differentiation of iPSCs into ECs *in vitro* and *in vivo*.** *A–I*, iPSCs differentiated for 4 days in presence of VEGF were transfected with miR-21 inhibitor (LNA-21) and the negative control (LNA-Ctrl). *A–C*, representative images of *in vitro* angiogenesis assay (*scale bar*, 25 μm) (*A* and *B*) and tube length quantification (*C*). *D–I*, representative images (*scale bar*, 100 μm) of *in vivo* angiogenesis assay and quantification of capillaries positive for the endothelial markers VE-cadherin (*VE-cad*) (*D–F*) and CD31 (*G–I*). The data presented are representative of means ± S.E. (*error bars*) of three independent experiments. *, *p* < 0.05; **, *p* < 0.01; ***, *p* < 0.001.

Next, miR-21 transfected cells were tested in *in vitro* and *in vivo* angiogenesis assays. Overexpression of miR-21 induced the formation of tubelike structures *in vitro* (Fig. 3, *A–C*). Inclusion of miR-21-overexpressing cells in *in vivo* Matrigel plugs led to the formation of a significantly higher number of CD31^+^ and VE-cadherin^+^ capillaries, as compared with the plugs seeded with precursor control transfected cells (Fig. 3, *D–I*). Accordingly, miR-21 inhibition reduced the number of capillary structures both *in vitro* (Fig. 4, *A–C*) and *in vivo* (Fig. 4, *D–I*). These results suggest that miR-21 is able to mediate a lineage-specific effect on iPSCs treated with VEGF to boost their differentiation into functional ECs.

##### Identification of the Molecular Targets of miRNA-21: TGF-β2 Pathway Is a Downstream Target of miRNA-21 and Drives iPSC Differentiation into ECs

Next, we aimed at elucidating the molecular mechanisms involved in miR-21-mediated differentiation of iPSCs. *In silico* predicted targets of miR-21 included some proteins involved in the angiogenic process. Transfected cells were analyzed for the expression of genes known to regulate EC functions and vessel growth, such as transforming growth factor β receptor II (*TGF*-β*RII*), regulator of ERK activation Sprouty 1 (*SPRY1*), RhoGTPase *RhoB* and *Sox7*, and genes encoding regulators of cell migration such as *vinculin*. It has been previously shown that in human adipose tissue-derived mesenchymal stem cells miR-21 directly targets the 3′-UTR of *TGF*-β*RII*, thus inducing adipogenic differentiation (17) or inhibiting tumor angiogenesis induced by the cells (15). In particular, increased expression of miR-21 in hADSCs inhibits VEGF up-regulation induced by TGF-β and therefore angiogenesis (15). On the contrary, *TGF*-β*RII* gene expression was not decreased by miR-21 overexpression or increased by its inhibition (data not shown). This result indicates that *TGF*-β*RII* is not directly targeted by miR-21 in ECs derived from iPSCs. Furthermore, we tested expression of genes of the Wnt and the HIF-1α/VEGF signaling pathways, which are known regulators of EC differentiation during embryogenesis and tumor growth (29–31) (data not shown). Our results demonstrated that *VEGF* expression was not inhibited by miR-21 overexpression or increased by its inhibition (data not shown); therefore, *VEGF* is not a direct target of miR-21 during the endothelial differentiation of iPSCs.

**FIGURE 5.**
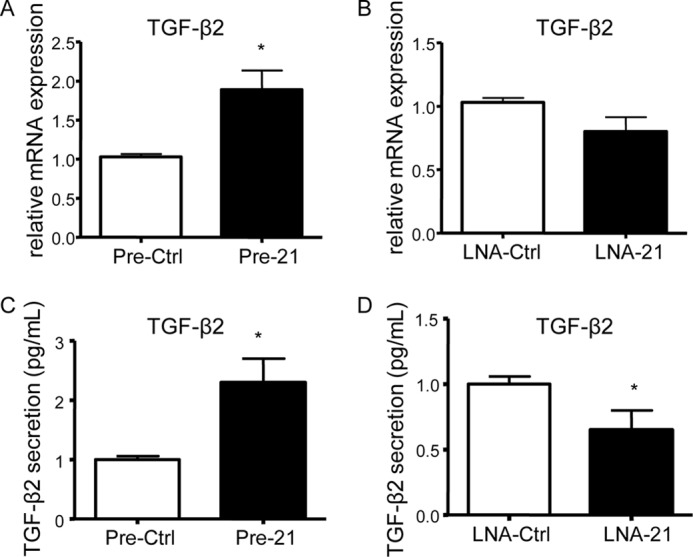
**TGF-β2 is a downstream target of miR-21.** *A–D*, iPSCs differentiated for 3 days with VEGF were transfected with miR-21 precursor (Pre-21), inhibitor (LNA-21), and the negative controls of the miR-21 precursor (Pre-Ctrl) and inhibitor (LNA-Ctrl), followed by quantitative RT-PCR assay (*A* and *B*) and ELISAs (*C* and *D*) 48 h after transfection to assess TGF-β2 expression and secretion levels, respectively. The data presented are representative of means ± S.E. (*error bars*) of three independent experiments. *, *p* < 0.05.

Additionally, we investigated the expression pattern of other genes belonging to the TGF-β family and found that *TGF*-β*2* was up-regulated by miR-21 overexpression (Fig. 5*A*), although not significantly down-regulated by miR-21 inhibition (Fig. 5*B*). Furthermore, ELISA result showed increased levels of secreted TGF-β2 in cells transfected with Pre-21 and reduced in cells treated with miR-21 inhibitor (Fig. 5, *C* and *D*).

**FIGURE 6.**
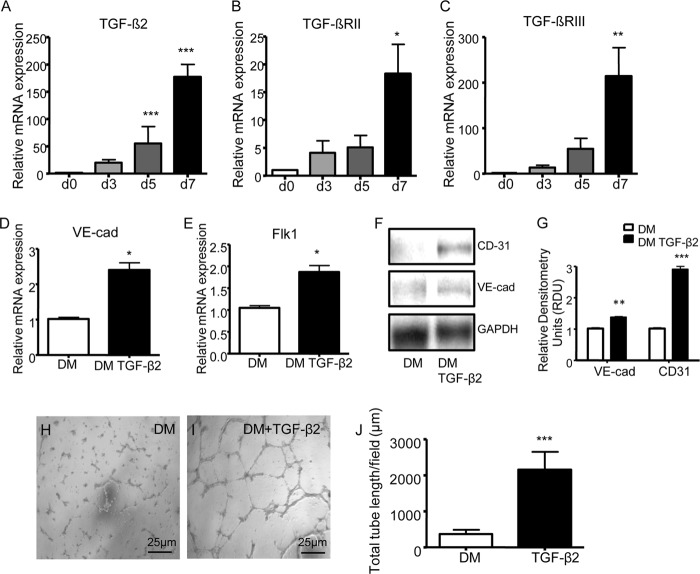
**TGF-β2 is increased in ECs derived from iPSCs and promotes this process.** *A–C*, iPSCs were differentiated with VEGF for up to 7 days, followed by quantitative RT-PCR assay to assess the expression of *TGF*-β*2* (*A*), *TGF*-β*RII* (*B*), and *TGF*-β*RIII* (*C*). *D–L*, iPSCs were treated for 7 days with 3 ng/ml TGF-β2 (DM + TGF-β2) or not (DM), followed by quantitative RT-PCR assay for the endothelial markers *VE-cadherin* and *Flk1* (*VE-cad*, *D* and *E*), Western blotting for the endothelial markers VE-cadherin (*VE-cad*) and CD31 (*F*, representative image; *G*, quantitative analysis), *in vitro* angiogenesis assay (representative images; *scale bar*, 25 μm) (*H* and *I*), and quantification of tube formation (*J*). The data presented are representative of means ± S.E. (*error bars*) of three independent experiments. *, *p* < 0.05; **, *p* < 0.01; ***, *p* < 0.001.

Analysis of *TGF*-β*2* expression in ECs derived from in iPSCs showed a strong and significant induction of *TGF*-β*2* gene expression, after 7 days of differentiation (Fig. 6*A*). This increase was matched by the increase of its receptors *TGF*-β*RII* and *III* (Fig. 6, *B* and *C*). Treatment of iPSCs with TGF-β2 induced a statistically significant up-regulation of *VE-cadherin* and *Flk1* expression (Fig. 6, *D* and *E*). Western blotting confirmed these results for CD31 and VE-cadherin at the protein level (Fig. 6, *F* and *G*). Moreover, iPSCs treated with TGF-β2 for 7 days showed a significantly increased tube formation capacity, as compared with untreated cells (Fig. 6, *H–J*). Importantly TGF-β2 treatment did not affect the smooth muscle marker gene expression (data not shown).

**FIGURE 7.**
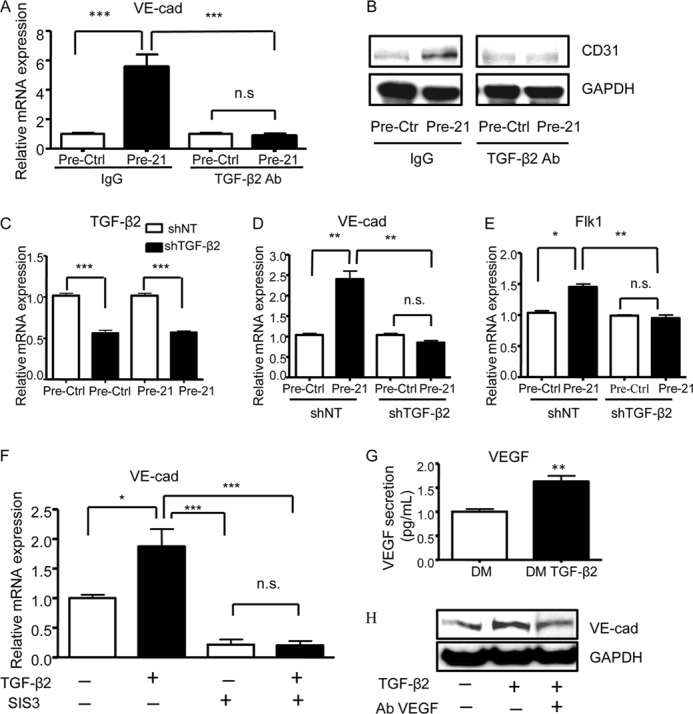
**The effect of miR-21/TGF-β2 on EC differentiation is mediated by SMAD3 and VEGF secretion.** *A* and *B*, iPSCs transfected with miR-21 precursor (Pre-21) and precursor control (Pre-Ctrl) after 3 days of differentiation in presence of VEGF were treated with 1 μg/ml TGF-β2 neutralizing antibody (TGF-β2 Ab) or IgG as a control; quantitative RT-PCR assay (*A*) and Western blotting (*B*) were performed 5 h after transfection to assess the expression levels of the EC markers *VE-cadherin* (*VE-cad*) and CD31, respectively. *C–E*, iPSCs transfected with miR-21 precursor (Pre-21) and precursor control (Pre-Ctrl) after 3 days of differentiation in presence of VEGF were infected with shRNA specific for *TGF*-β*2* (shTGF-β2) or nontargeting shRNAs (shNT) as a control; quantitative RT-PCR assay was performed 48 h after infection to assess the expression of *TGF*-β*2* (*C*), *VE-cadherin* (*VE-cad*) (*D*), and *Flk1* (*E*). *F*, iPSCs differentiated with (+) or without (−) TGF-β2 for 7 days were treated with SMAD3 inhibitor (SIS3) or DMSO as control, followed by quantitative RT-PCR assay to assess the expression of *VE-cadherin* (*VE-cad*). *G*, ELISAs showing VEGF secretion in iPSCs treated for 7 days with TGF-β2. *H*, iPSCs differentiated with (+) or without (−) TGF-β2 for 7 days were simultaneously treated with VEGF neutralizing antibody (Ab VEGF +) or IgG as a control (Ab VEGF −), followed by Western blotting to assess VE-cadherin (*VE-cad*) expression. The data presented are representative of means ± S.E. (*error bars*) of three independent experiments. *, *p* < 0.05; **, *p* < 0.01; ***, *p* < 0.001.

To confirm the contribution of TGF-β2 in mediating miR-21 effect on EC differentiation, Pre-21 transfected iPSCs were treated with TGFβ-2 neutralizing antibody. TGF-β2 neutralization reduced the miR-21-induced VE-cadherin and CD31 expression at both gene and protein expression levels (Fig. 7, *A* and *B*). To further confirm this result, we established a stable knockdown of *TGF*-β*2* in iPSCs differentiated for 3 days in presence of VEGF, using specific shRNAs. Following the knockdown, cells were transfected with Pre-21, and EC marker expression was analyzed. *TGF*-β*2* gene expression showed a significant repression after infection with shTGF-β2, confirming the efficiency of the shRNA knockdown of *TGF*-β*2* (Fig. 7*C*). Importantly, in Pre-21 transfected cells, the gene expression levels of *VE-cadherin* and *Flk1* showed a significant reduction after shTGF-β2 infection (Fig. 7, *D* and *E*). Remarkably, to our knowledge, these findings are the first to suggest a role for TGF-β2 in the miR-21-mediated endothelial differentiation of iPSCs.

To clarify the molecular mechanisms through which TGF-β2 is able to drive iPSC differentiation into ECs, we focused on the role of the TGF-β2/SMAD3 pathway, which has been previously shown to mediate TGF-β-dependent ES cell differentiation (32). Treatment of iPSCs with the SMAD3 inhibitor SIS3 significantly reduced *VE-cadherin* expression, in the presence or absence of TGF-β2 (Fig. 7*F*). Furthermore, we analyzed the effect of TGF-β2 treatment on VEGF levels, based on previous studies showing that VEGF secretion is induced by TGF-β2 (33, 34). ELISAs performed on iPSCs treated with TGF-β2 for 7 days showed a significant induction in the VEGF secretion, as compared with untreated cells (Fig. 7*G*). Additionally, in iPSCs differentiated for 7 days with TGF-β2 treatment, VEGF-blocking antibody reduced the TGF-β2-dependent EC marker expression (Fig. 7*H*). These data suggest that TGF-β2 induces EC differentiation in iPSCs through SMAD-dependent pathway and by inducing VEGF secretion. However, TGF-β2 treatment of iPSCs was not able to induce miR-21 up-regulation (data not shown). The result indicates that TGF-β2 does not act upstream of miR-21. These findings could indicate that additional pathways may also act together with TGF-β2 to contribute to VEGF secretion, leading to maintenance of endothelial differentiation state.

##### Identification of the Molecular Targets of miRNA-21: miR-21 Targets the PTEN/Akt Pathway, Which Regulates iPSC Differentiation into ECs

**FIGURE 8.**
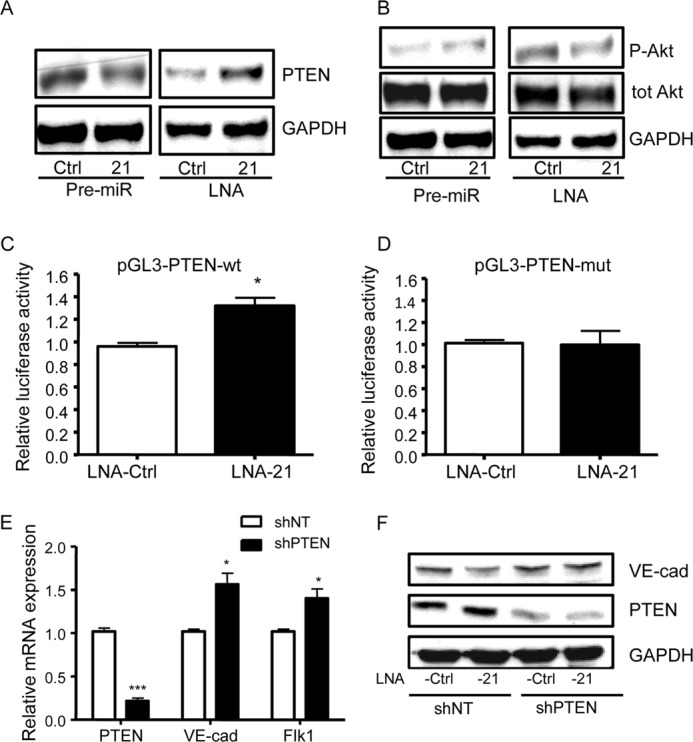
**miR-21 targets the PTEN/Akt pathway, which regulates iPSC differentiation into ECs.** *A* and *B*, iPSCs differentiated for 3 days with VEGF were transfected with miR-21 precursor (Pre-21), inhibitor (LNA-21), and the negative controls of the miR-21 precursor (Pre-Ctrl) and inhibitor (LNA-Ctrl), followed by Western blotting 48 h after transfection to assess PTEN expression (*A*) and Ser(P)-473 Akt (*P-Akt*) and total Akt (*tot Akt*) expression (*B*). *C* and *D*, the wild type or mutated reporter plasmid pGL3 for *PTEN* 3′-UTR (pGL3-PTEN-wt, *C* or pGL3-PTEN-mut, *D*) was co-transfected with miR-21 inhibitor (LNA-21) and its control (LNA-Ctrl) in iPSCs differentiated for 3 days with VEGF, followed by luciferase assay 48 h after transfection. *E*, iPSCs differentiated for 3 days with VEGF, were infected with shRNA specific for *PTEN* (shPTEN) or nontargeting shRNAs (shNT) as a control, followed by quantitative RT-PCR assay to assess the expression of *PTEN*, *VE-cadherin* (*VE-cad*), and *Flk1* 48 h after infection. *F*, iPSCs transfected with miR-21 inhibitor (LNA-21) and inhibitor control (LNA-Ctrl) after 3 days of differentiation in the presence of VEGF were infected with shRNA specific for *PTEN* (shPTEN) or nontargeting shRNAs (shNT) as a control; Western blotting was performed 48 h after infection to assess the expression of PTEN and VE-cadherin (*F*). The data presented are representative of means ± S.E. (error bars) of three independent experiments. *, *p* < 0.05; ***, *p* < 0.001.

One of the *in silico* predicted targets for miR-21 is phosphatase and tensin homolog (*PTEN*). Recent studies indicated that miR-21 inhibited the tumor suppressor *PTEN* by binding to its 3′-UTR (35). Inhibition of *PTEN* by miR-21 has been reported to induce tumor angiogenesis through Akt and ERK activation and HIF-1α expression (13). Moreover, miR-21 has been shown to target PTEN/Akt and partly mediate TGF-β-induced endothelial to mesenchymal transition (36). However, so far the role of PTEN in miR-21-mediated endothelial differentiation of iPSCs has not been elucidated. We first confirmed *PTEN* as a direct target of miR-21 at a protein expression level. In iPSCs differentiated with VEGF for 3 days, transfection with Pre-21 caused a significant reduction of PTEN protein expression, whereas transfection with LNA-21 led to a significant induction of PTEN (Fig. 8*A*). Because PTEN is known to be the antagonist of PI3K, which removes the 39 phosphate of phosphatidylinositol 1,4,5-trisphosphate, resulting in inhibition of the Akt signaling pathway (37), we aimed to assess whether PTEN inhibition is required for the activation of Akt during the miR-21-mediated iPSC differentiation process. Concomitantly, previous data from our group showed that the PI3K/Akt signaling pathway drives the shear-induced ES cell differentiation into ECs (24). Our results show that miR-21 overexpression increased the phosphorylation of Akt at the serine 473 site, whereas inhibition of miR-21 reduced Akt phosphorylation, as compared with the relative negative controls. The total level of Akt was not significantly altered by miR-21 overexpression or inhibition (Fig. 8*B*). In parallel, to further confirm *PTEN* as a direct target of miR-21, we performed a luciferase assay to assess the binding of miRNA to mRNA. Co-transfection of the WT *PTEN* 3′-UTR plasmid and LNA-21 in iPSCs differentiating with VEGF resulted in a significant increase in luciferase activity (Fig. 8*C*). Importantly, mutations in the sequence targeted by miR-21 in *PTEN* 3′-UTR abolished the observed up-regulation of luciferase activity by miR-21 (Fig. 8*D*). These results confirmed *PTEN* as a direct target of miR-21 and validated its specific binding to the predicted binding site.

We therefore decided to assess whether the PTEN/Akt pathway was essential in driving miR-21-mediated iPSC differentiation into EC. We indeed aimed to establish a stable knockdown of *PTEN* in iPSCs differentiated with VEGF using specific shRNAs, to then analyze the EC marker expression. *PTEN* gene expression showed a significant repression in iPSCs infected with shPTEN, confirming the efficiency of the shRNA knockdown of *PTEN*. Importantly, *VE-cadherin* and *Flk1* showed a statistically significant increase in the gene expression after shPTEN infection (Fig. 8*E*). We then analyzed the expression of the EC markers in iPSCs treated with VEGF and transfected with LNA-21, using shPTEN. PTEN protein expression showed a significant down-regulation in iPSCs infected with shPTEN, confirming the efficiency of the shRNA knockdown of *PTEN* (Fig. 8*F*). Our results also showed that in LNA-21 transfected cells, the protein levels of VE-cadherin were significantly increased after shPTEN infection (Fig. 8*F*).

These data suggest that during EC differentiation of iPSCs, miR-21 inhibits PTEN expression, which in turn increases Akt phosphorylation. The results presented in this study also highlight that PTEN inhibition is required for miR-21 mediated up-regulation of EC markers in differentiated iPSCs.

## DISCUSSION

Regeneration of damaged vessels and promotion of new vessel formation in infarcted tissues require the identification of a suitable source of ECs that can be derived from the patient in adequate number for clinical use. Somatic cells from patients have the potential to be reprogrammed to iPSCs, which can be expanded in an undifferentiated state, or to be subjected to lineage specific differentiation in response to a stimulus. In the present study, we established a differentiation protocol for iPSCs, based on the use of collagen IV and 50 ng/ml VEGF. We demonstrated that miR-21 is overexpressed in ECs derived from iPSCs, as compared with the undifferentiated cells in gelatin, and mediates endothelial differentiation through targeting-specific pathways. Thus, our findings provide the basic information for production of endothelial cells from iPSCs that has a great potential in regenerative medicine.

Previously, Zeng *et al.* (24) stimulated Sca-1^+^ vascular progenitor cells with medium containing 10 ng/ml VEGF, and Narazaki *et al.* (38) cultivated Flk1^+^ progenitor cells with 100 ng/ml VEGF. In the present study, we tested several concentrations ranging between these two extremes by using undifferentiated iPSCs without predifferentiating the cells or selecting for a specific progenitor population. We established that the treatment of iPSCs with increasing concentrations of VEGF had an effect on endothelial differentiation up to 50 ng/ml, which led to a faster differentiation. Importantly, compared with previously described differentiation protocols, our protocol shows a higher scale of EC marker induction, up to a 100-fold increase in *Flk1* expression, thus indicating better differentiation efficiency. For instance, Narazaki *et al.* (38) showed that, in Flk1^+^ progenitor cells, the effect of VEGF only led to a 20-fold increase in EC marker expression. In conclusion, our protocol allows the direct and efficient EC differentiation from undifferentiated iPSCs in a relative short time and at the highest level, as compared with the previously published protocols.

One of the main aims in this study was to establish the molecular mechanisms driving the differentiation of iPSCs to ECs. Previous work on ES cells has shown that pluripotent cells and cells differentiated to vascular ECs express different pools of miRNAs (28). To our knowledge, there are no studies investigating the miRNA profile during vascular differentiation of iPSCs though recent publications (39). We decided to perform miRNA arrays on iPSCs differentiated for 3 days with VEGF and undifferentiated cells. We obtained a pool of differentially expressed miRNAs and validated five of the most interesting candidates. Among those, we decided to focus on miR-21, a molecule previously reported in tumor angiogenesis and adipogenic differentiation of stem cells (13–17). It was found that miR-21 could induce EC marker expression in iPSCs predifferentiated with VEGF, thus boosting the efficiency of differentiation. These results were confirmed at a protein level and through *in vivo* and *in vitro* functional angiogenesis assays. In the study of Liu *et al.* (13), Matrigel plugs seeded with Pre-21- and LNA-21-transfected human prostate cancer cells were applied onto the chicken chorioallantoic membrane of 9-day-old embryos to assess tumor angiogenesis. Supporting our findings, the number of branches of microvessels were increased by miR-21 overexpression and vice versa decreased by miR-21 inhibition, indicating a pro-angiogenic function of miR-21. In our study, the *in vivo* angiogenesis process appears to be promoted by miR-21 transfected cells. These results suggest that miR-21 is a key modulator in the endothelial differentiation of iPSCs. The screening for miR-21 potential targets is a challenging process, because prediction algorithms generate a high percentage of false positives.

We showed that miR-21 induced TGF-β2 expression and secretion during EC differentiation. Importantly, TGF-β2 neutralization and knockdown inhibited the miR-21-mediated EC marker induction, thus indicating the requirement of TGF-β2 during this process. Accordingly, expression of *TGF*-β*2* and its receptors appeared to be highly increased in iPSCs differentiated with VEGF. TGF-β2 secretion from iPSCs transfected with Pre-21 appeared to be only in the scale of pg/ml. However, because iPSCs continuously secrete TGF-β2, which probably exerts a paracrine action on neighboring cells, that concentration was sufficient to be effective in iPSC differentiation.

TGF-β has been so far shown to induce smooth muscle differentiation via Notch or SMAD2 and SMAD3 signaling in ES cells (32) or in a neural crest stem cell line (40). Here, we report that TGF-β2 treatment is able to induce iPSC differentiation specifically toward EC lineage, without affecting smooth muscle gene expression. TGF-β has been shown to bind to TGF-βRI and to induce phosphorylation of SMAD2/3, thereby inhibiting proliferation, tube formation, and migration of ECs (41). However, so far there are no studies reporting the role of SMAD3 in stem cell differentiation into ECs. The results reported in the present study suggest the involvement of the TGF-β2/SMAD3 pathway in the iPSC differentiation process; SMAD3 inhibition not only abolished TGF-β2 induction of EC markers, but also reduced the baseline expression of *VE-cadherin*, in the absence of TGF-β2 stimulation. This result may be explained by considering that SMAD3 is at the center of many other molecular pathways, and it is not only a TGF-β2 downstream effector. For instance, it has been reported that SMAD2/3 is a downstream effector of Activin/Nodal signaling that shares some biological activities with TGF-β (42); moreover, SMAD2/3 signaling has been shown to keep vascular integrity through regulating N-cadherin, VE-cadherin, and S1PR1 expression (43).

miR-21 has been previously shown to target *PTEN* through Akt and ERK activation to induce tumor angiogenesis (13) and through Akt to partly mediate TGF-β-induced endothelial to mesenchymal transition (36). Our findings showed that miR-21 directly and specifically targets the PTEN/Akt pathway in EC differentiation derived from iPSCs. Interestingly, it has been shown that the PI3K/Akt signaling pathway plays a crucial role in many intracellular cascade events including tumor angiogenesis and tumor growth (44), and in particular, the PI3K/Akt pathway has been reported to drive the shear-induced stem cell differentiation into ECs (24). These results highlight that *PTEN* knockdown is required to induce EC marker expression in iPSCs differentiated to ECs and transfected with miR-21 inhibitor.

In a recently published study, it has been shown that Akt-mediated Twist1 phosphorylation promotes epithelial-mesenchymal transition and breast cancer metastasis by modulating its transcriptional target TGF-β2; this leads to enhanced TGF-β receptor signaling, which in turn maintains hyperactive PI3K/Akt signaling (45). It is therefore possible that miR-21 activation of the PTEN/Akt pathway may induce Twist1 phosphorylation. Activation of Twist1 may target and induce TGF-β2 to mediate the endothelial differentiation of iPSCs. Phosphorylated Twist1 may indeed have a key role in mediating cross-talk between the miR-21/Akt and TGF-β/Smad signaling axes that supports endothelial differentiation of iPSCs. Other additional pathways might be also involved in this process and will be investigated in future.

**FIGURE 9.**
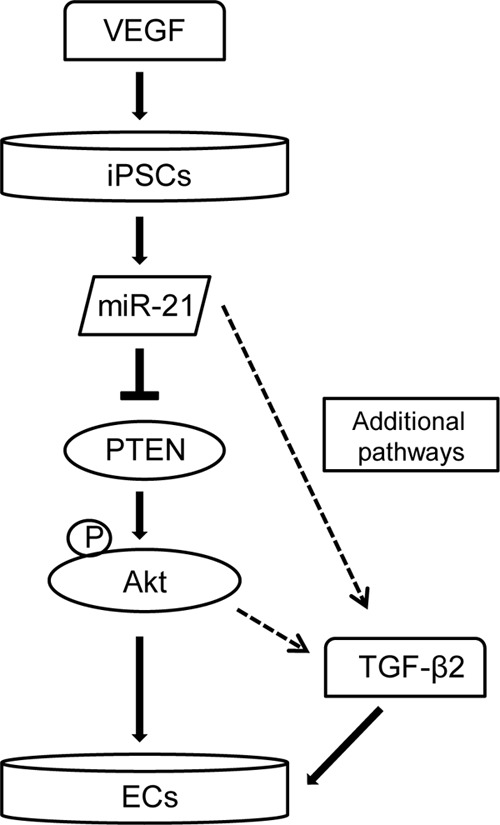
**Schematic representation of the mechanisms of iPSC differentiation into ECs.** In ECs, differentiation derived from iPSCs VEGF induces miR-21, which targets PTEN/Akt and results in TGF-β2 activation and robust induction of EC differentiation.

In summary, the data presented in this study suggest that in iPSCs predifferentiated with VEGF, miR-21 targets PTEN/Akt and induces TGF-β2, therefore mediating endothelial differentiation (Fig. 9). Indeed, we established a new link between VEGF, miR-21, and TGF-β2 during the endothelial differentiation of iPSCs. Elucidation of the molecular pathways involved in the miR-21-mediated iPSC differentiation into ECs might provide the basic information for stem cell therapy of vascular diseases, *e.g.*, tissue engineering and endothelial repair in damaged vessels.

## Footnotes

3 The abbreviations used are:

EC: endothelial cell
iPSC: induced pluripotent stem cell
miRNA/miR: microRNA
LNA: locked nucleic acid
shPTEN: PTEN shRNA lentivirus
shNT: nontarget shRNA lentivirus
HIF: hypoxia-inducible factor
hADSC: human adipose-derived stem cell
DM: differentiation medium
TGF-βRII: transforming growth factor β receptor II.
